# The LC-MS/MS-Based Measurement of Isopimaric Acid in Rat Plasma and Application of Pharmacokinetics

**DOI:** 10.1155/2021/2310422

**Published:** 2021-10-15

**Authors:** Doudou Huang, Jiaxi Cheng, Junqin Mao, Senlin Ma, Zenan Du, Wansheng Chen, Feng Zhang, Lianna Sun

**Affiliations:** ^1^Department of TCM Processing, Shanghai University of Traditional Chinese Medicine, Shanghai 201203, China; ^2^Institute of Chinese Materia Madica, Shanghai University of Traditional Chinese Medicine, Shanghai 201203, China; ^3^Department of Clinical Pharmacy, Shanghai Jiangqiao Hospital Jiading Branch, Shanghai 201803, China; ^4^Emergency Department, Huashan Hospital, Fudan University, Shanghai 200040, China; ^5^Department of Pharmacy, Changzheng Hospital, Navy Military Medical University, Shanghai 200433, China

## Abstract

Isopimaric acid (IPA) exhibits a diverse array of pharmacological activities, having been shown to function as an antihypertensive, antitumor, antibacterial, and hypocholesterolemic agent. However, few studies of the pharmacokinetics of IPA have been performed to date, and such analyses are essential to explore the in vivo mechanisms governing the biological activity of this compound. As such, we herein designed a selective LC-MS approach capable of quantifying serum IPA levels in model rats using an Agilent HC-C18 column (250 mm × 4.6 mm, 5 *μ*m) via isocratic elution with a mobile phase composed of methanol 0.5% formic acid (91 : 9, v/v) at a 1 mL/min flow rate. Ion monitoring at m/z 301.2 [M-H]^−^ was used to quantify IPA levels in plasma samples from these rats, while internal standard (IS) levels were assessed at m/z 455.3 [M-H]^−^. After validation, this approach was employed to conduct a pharmacokinetic analysis of rats administered IPA via the oral (p.o. 50, 100, or 200 mg/kg) and intravenous (i.v. 5 mg/kg) routes. Analyses of noncompartmental pharmacokinetic parameters revealed that IPA underwent secondary absorption following oral administration to these animals, with the two tested oral doses (50 and 100 mg/kg) being associated with respective absolute bioavailability values of 11.9% and 17.5%. In summary, this study may provide a foundation for future efforts to explore the mechanistic basis for the pharmacological activity of IPA, offering insights to guide its subsequent clinical utilization.

## 1. Introduction

Rosin acids including isopimaric acid (IPA), sandaracopimaric acid, and pimaric acid are major bioactive derivatives of *Pinus* genus member plants. Pharmacological activities of these acids have shown them to exhibit diverse effects including hypocholesterolemic activity [1], antigastric ulcer activity [2], blood glucose lowering activity [3], and macrocyte clumping activity [4]. IPA is a diterpenoid containing a double bond and methyl and ethenyl residues at C-12 that has previously been reported to exhibit antibacterial [5], antitumor [6, 7], and hypocholesterolemic activity [8], in addition to being able to prevent high blood pressure via the activation of a large conductance Ca^2+^ and K^+^ channel [9, 10]. Preparations containing this compound, including Kanglao, Cebai NO.V, and IPA capsules, are often used to treat pulmonary tuberculosis patients in Chinese clinics, wherein they have been found to exhibit marked efficacy against this disease. IPA is the primary bioactive compound in all three of these preparations, emphasizing its pharmacological utility [11, 12].

Pharmacokinetic profiling is vital to effective drug development. To date, the only published reports regarding IPA quantification have focused on employing an HPLC approach to measure the levels of this acid in herbal medicine preparations using a methanol:water:acetic acid (60 : 37 : 3; v:v) mobile phase [12]. No studies have reported sensitive assays capable of analyzing IPA levels or pharmacokinetics in biological fluid samples. The development of such an assay, however, is essential to facilitate the clinical study of IPA pharmacokinetic and pharmacodynamic properties.

LC-MS techniques combine the high-efficiency separation of LC with the high sensitivity and structural characterization abilities of MS, making these methods ideal for assessing the levels of particular compounds of interest in plants of biofluids [13–19]. Herein, we thus designed a sensitive and specific LC-MS/MS approach to quantifying IPA concentrations in rat plasma. After validating the accuracy, precision, sensitivity, and specificity of this approach, we applied it to study the pharmacokinetics of IPA following its oral and i.v. administration in rats.

## 2. Materials and Methods

### 2.1. Reagents

IPA (purity >98%) and betulinic acid (internal standard, IS; purity >98%) were, respectively, from Shanghai Jianglai Biological Technology Co., Ltd. (Shanghai, China) and the National Institute for the Control of Pharmaceutical and Biological Products (Beijing, China). Burdick & Jackson (Ulsan, Korea) provided HPLC grade methanol, whereas Anaqua Chemical Supply Company (TX, USA) was the source of HPLC grade formic acid. All other reagents were of analytical grade.

### 2.2. Animals

Male Sprague-Dawley rats (220-250 g) from Shanghai Slac Laboratory Animal Co., Ltd. (Shanghai, China) (2018(hu)-08-0013) were housed in a climate-controlled facility (22 ± 2°C, 46 ± 20% relative humidity, 12 h light/dark cycle) with unlimited food and water access. After a 7-day acclimatization period, 20 rats were randomly assigned to undergo the administration of IPA via p.o. administration (50, 100, or 200 mg/kg) or via i.v. injection (5 mg/kg) for pharmacokinetic analyses. All animal studies received approval from the Animal Ethics Committee of the Naval Military Medical University.

### 2.3. LC-MS/MS Conditions

An Agilent HC-C_18_ column (250 mm × 4.6 mm, i.d. 5 *μ*m) and an Agilent SB C_18_ guard column (12.5 mm × 4.6 mm, i.d. 5 *μ*m) were used to achieve chromatographic separation with the Agilent 1100 HPLC system (CA, USA) using a degasser (G1322A), quaternary pump (G1311A), well-plate autosampler (G1367A; kept at 4°C and programmed to draw 40 *μ*L sample aliquots), and a thermostated column compartment (G1316A). The isocratic mobile phase was composed of methanol (A) and 0.5% formic acid in water (B) at a 91 : 9 ratio, with a flow rate of 1 mL/min. Columns were kept at 30°C, and each sample was run for 13 min.

An Agilent G1946D MS instrument with an ESI source was used to detect IS and IPA in samples. A 1 : 4 postcolumn split ratio was applied, with the mass spectrometer being directly connected to the flow outlet using a three-way joint. The following electrospray ionization conditions were utilized: a capillary voltage of 3500 V, nebulizer pressure of 40 psi, drying gas at 10 L/min at 350°C gas, and fragmentor voltage of 100 V. Samples were quantitatively assessed in negative ion mode with select ion monitoring (SIM), with IPA and IS being recorded at m/z 301.2([M-H]^−^) and m/z 455.4([M-H]^−^).

### 2.4. Calibration and Quality Control Sample Preparation

Methanol was used to prepare a 1.001 mg/mL IPA stock solution that was diluted in methanol to yield working standard samples that were subsequently used to prepare assay standards by adding 20 *μ*L of working standards to 100 *μ*L of blank rat plasma, yielding samples with IPA concentrations of 4, 10, 50, 100, 400, 1000, 2000, and 4000 ng/mL. Quality control (QC) samples were prepared at three concentration levels (10, 400, and 2000 ng/mL) via the same approach. A working IS solution was prepared by using methanol to dilute an 850.0 *μ*g/mL stock solution to 85 *μ*g/mL. These samples were prepared for each analysis, were maintained at 4°C, and were used after warming to room temperature.

### 2.5. Sample Preparation

Samples were prepared by extracting IPA via combining 100 *μ*L rat plasma samples with 20 *μ*L of IS and 1 mL of a mixture of ethyl acetate: *n*-hexane (4 : 1). Samples were then vortexed in 1.5 mL polypropylene tubes for 1 min and spun down for 5 min at 1,500 × *g* at 4°C, and 800 *μ*L supernatant aliquots were transferred to fresh tubes and evaporated until dry using a speed vac concentrator. The remaining residue was then resuspended in 50 *μ*L methanol, vortexed for 1 min, and spun down for 5 min at 9,600 × g. A 40 *μ*L supernatant aliquot from this sample was then injected for LC-MS/MS assessment.

### 2.6. Methodological Validation

#### 2.6.1. Selectivity Analysis

Chromatograms for six separate blank plasma samples were compared to corresponding chromatograms from plasma sample spiked with IS and IPA at defined concentrations or with plasma samples from rats collected following IPA administration in order to assess LC-MS/MS analytical selectivity.

#### 2.6.2. Linearity and Detection Limits

In order to establish calibration curves, analyte-to-IS peak area ratios were plotted against plasma concentrations with a 1*/x* weighted linear least squares regression model. IPA quantification linearity was evaluated using five independent analytical runs assessing eight standards containing different IPA concentrations, with a correlation coefficient > 0.99 being indicative of satisfactory linearity.

The lowest IPA concentration yielding a signal − to − noise ratio (SNR) ≥ 3 was defined as the lower limit of detection (LLOD), while the lowest concentration with an SNR ≥ 5 that could reliably be quantified with 80-120% accuracy and <20 precision was defined as the lower limit of quantification (LLOQ).

#### 2.6.3. Precision and Accuracy

Precision and accuracy were evaluated by assessing five replicate QC samples at the three concentration levels within the assay linear range, with the resultant values being, respectively, expressed as relative standard deviation (RSD) and relative error (RE) values. Accuracy and precision were measured on three consecutive days to establish the consistency of these measures. An RSD < 15% and an RE of ±15% were considered indicative of satisfactory precision and accuracy, respectively.

#### 2.6.4. Extraction Recovery and Matrix Effects

IPA extraction recovery was assessed using five replicate samples at the three QC concentration levels by examining differences in the response ratios for extracted plasma QC samples and those of extracted blank plasma spiked to contain an identical IPA concentration. Absolute matrix effects were defined by evaluating relative peak areas of analytes in the extracted blank plasma and comparing them to those of extracted methanol. Relative matrix effects were determined based upon the RSD of the mean peak areas of the analytes in the extracted blank plasma. Matrix effects were considered to be negligible if analyte peak area ratios were from 85-115%.

#### 2.6.5. Stability

IPA stability was examined by evaluating five replicate samples at the three QC concentration levels in a range of storage conditions. To measure short-term stability, samples were maintained at room temperature for 2 h. Processed sample stability was measured by incubating them for 24 h in the room temperature autosampler. In addition, long-term stability was measured following a 30-day storage period at -20°C, and freeze-thaw stability was measured by cycling samples from -20°C to 20°C three times. When the assay values associated with these samples were within a ±15% deviation from the nominal value, samples were considered to be stable.

### 2.7. Pharmacokinetic Study

After developing and validating the above LC-MS/MS approach, it was employed to assess the pharmacokinetics of IPA following its intravenous or oral administration to rats as per the approved protocols discussed above. Briefly, after acclimatization, rats were fasted for 12 h and then randomized to undergo treatment with a 5 mg/kg i.v. dose of IPA or with oral IPA doses of 50, 100, or 200 mg/kg (*n* = 5/group). All IPA solutions were prepared in physiological saline and thoroughly mixed with CMC-Na. Blood samples (~0.4 mL) from these rats were collected in heparinized tubes at 0, 0.083, 0.167, 0.25, 0.5, 0.75, 1, 2, 3, 4, 6, 8, and 12 h following i.v. injection or at 0.167, 0.333, 0.5, 0.75, 1,1.5, 2, 4, 6, 8, 12, and 24 h following oral administration. Blood samples were spun for 5 min at 1,500 × g, after which plasma was collected and stored at -20°C prior to analysis.

### 2.8. Data Analysis

Plasma IPA concentrations were determined using the calibration curve established above based on peak area ratios from individual injection samples. The mean residence time (MRT0-*t*), area under the blood concentration-time curve (AUC0-∞, AUC0-*t*), body clearance (CL/F), and elimination half-life (*T*_1/2_) were quantified with the DAS 3.2.6 program (Chinese Pharmacology Society) via a noncompartmental approach. Concentration-time curves were used to establish maximum concentration (*C*_max_) and time to maximum concentration (*T*_max_) values. Data are given as means ± standard deviation (S.D.). Absolute bioavailability (*F* %) was determined as follows: *F*% = AUC0 − ∞(p.o.) dose (i.v.)/AUC0 − ∞(i.v.) dose (p.o.) × 100%.

## 3. Results and Discussion

### 3.1. Methodological Development

**Figure 1:**
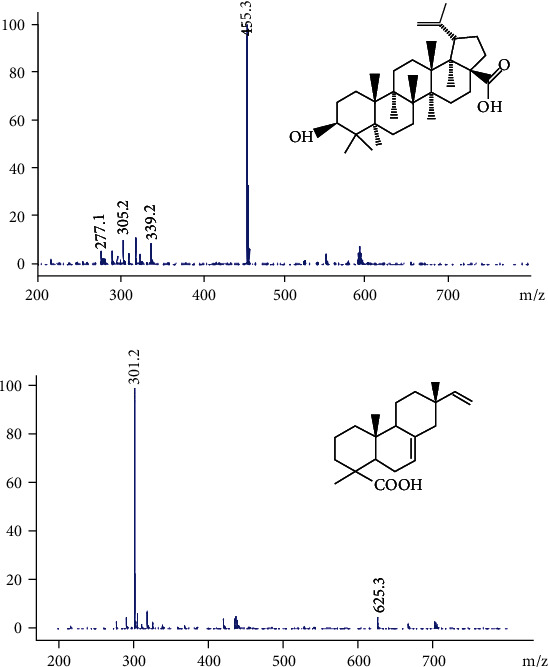
Protonated molecular ions in the mass spectra for IPA (a) and betulinic acid (b).

To develop an approach to quantifying plasma IPA levels, IPA responses to positive and negative electrospray ionization across the full-scan mass spectra were recorded, revealing better sensitivity in negative ion mode. IPA and IS mass scan spectra and corresponding chemical structures are shown in Figure 1. Quantitation was conducted by selecting the most abundant fragmentation ions in the spectra ([M-H]^−^ m/z 301.2 for IPA and [M-H]^−^ m/z 455.3 for IS). In addition, ESI parameters were optimized via flow injection analysis (FIA) using the IPA standards, with the following time segments being recorded: 8–11 min for IS m/z 455.3 and 11–14 min IPA at m/z 301.2.

A range of mobile phases were tested to optimize separation conditions, revealing that a mobile phase made of methanol:water:formic acid (91 : 9 : 0.5, v/v/v) was able to facilitate good IPA and IS retention and sensitivity.

A liquid-liquid extraction approach was selected because it excludes the potential adverse impacts of sodium and potassium on the instrument owing to their involatility, because it facilitates better extraction and a lower LLOQ than IPA protein precipitation approaches and because it allows for sample condensation, enabling the analysis of samples with concentration ranges between the LLOD and LLOQ that cannot otherwise be detected. A range of extraction solvents for IS and IPA were tested, including diethyl ether and ethyl acetate with or without *n*-hexane. While diethyl ether allowed for easy evaporation, it also often causes emulsification, interfering with IPA recovery and separation. Ethyl acetate enabled aqueous and organic phase separation, but the endogenous components interfered with the associated chromatographic peaks. This disturbance was eliminated when ethyl acetate and *n*-hexane were mixed at a 4 : 1 ratio.

### 3.2. Methodological Validation

#### 3.2.1. Selectivity

**Figure 2:**
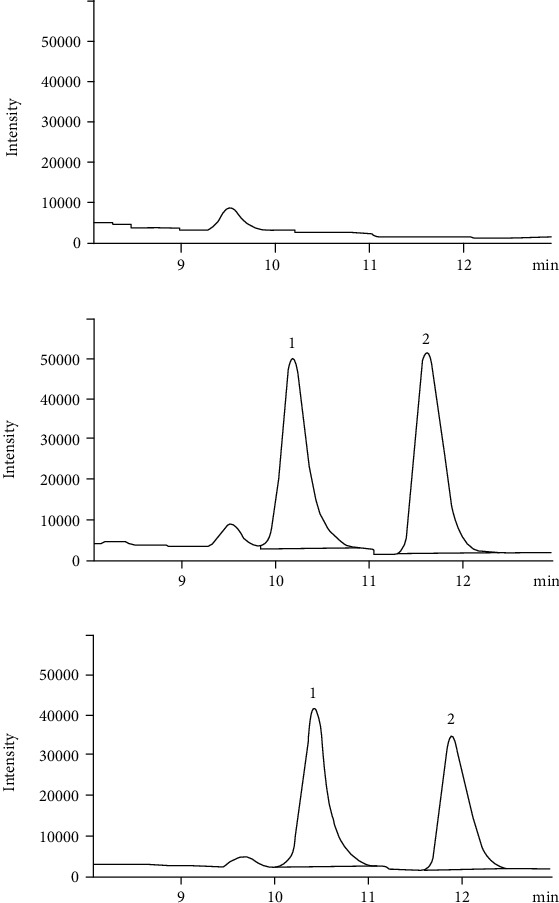
Comparative chromatograms of selectivity of betulinic acid (IS) and IPA of blank plasma (a), spiked standard solution in blank plasma (b), and rat plasma samples after administering (c). The peaks marked in chromatograms were as follows: (1): betulinic acid (IS) and (2): IPA.

IPA and IS representative chromatograms are shown in Figure 2, with these two compounds exhibiting respective retention times of approximately 11.8 min and 10.4 min. No interference from endogenous compounds was detected at these retention times.

#### 3.2.2. Linearity, LLOD, and LLOQ

Calibration curves for IPA remained linear from 4 to 4000 ng/mL, with a least squares regression equation of *y* = 1.548*x* − 0.0047 (*r* = 0.9987) and with deviations of <15% for all tested calibration concentrations. IPA had an LLOQ of 4 ng/mL and an LLOD of 2 ng/mL, with these values being sufficiently low to permit pharmacokinetic studies of IPA in rats.

#### 3.2.3. Precision and Accuracy

The results of IPA precision and accuracy analyses of five replicates of three QC sample levels are shown in Table 1. Intra- and interassay precision values for IPA were from 5.8-7.4% to 7.3-10.3%, respectively, while respective accuracy values ranged from -6.7 to 5.6% and -3.5 to 2.9%. These results indicated that this LC-MS/MS approach was a reliable means of reproducibly quantifying plasma IPA levels.

#### 3.2.4. Matrix Effects and Recovery

For IPA QC samples at the low, medium, and high concentrations, the matrix effect values were 131.5%, 129.0%, and 120.6%, respectively, with RSD values from 7.1 to 11.6% (Table 2), consistent with an absence of significant matrix effects under the tested conditions. IPA recovery rates ranged from 103.9 to 113.4% at these three QC levels (Table 2), which were met the requirements of Chinese Pharmacopoeia, in which the relative recovery rate measured at the high and medium concentration points should be 85~115%, and the low concentration should be 80~120%. Precision values corresponding to IPA recovery were all <9.5%, indicative of reproducible and consistent recovery.

#### 3.2.5. Stability

IPA stability in rat plasma samples was tested for three different QC sample concentrations as shown in Table 3, revealing that all samples remained stable in plasma following a 3 h room temperature incubation, 30 days of storage at -20°C, or 3 rounds of freeze-thawing. Postpreparation stability analyses similarly indicated that samples remained stable when stored at room temperature for 24 h in the autosampler.

#### 3.2.6. Analysis of the Pharmacokinetics of IPA

**Figure 3:**
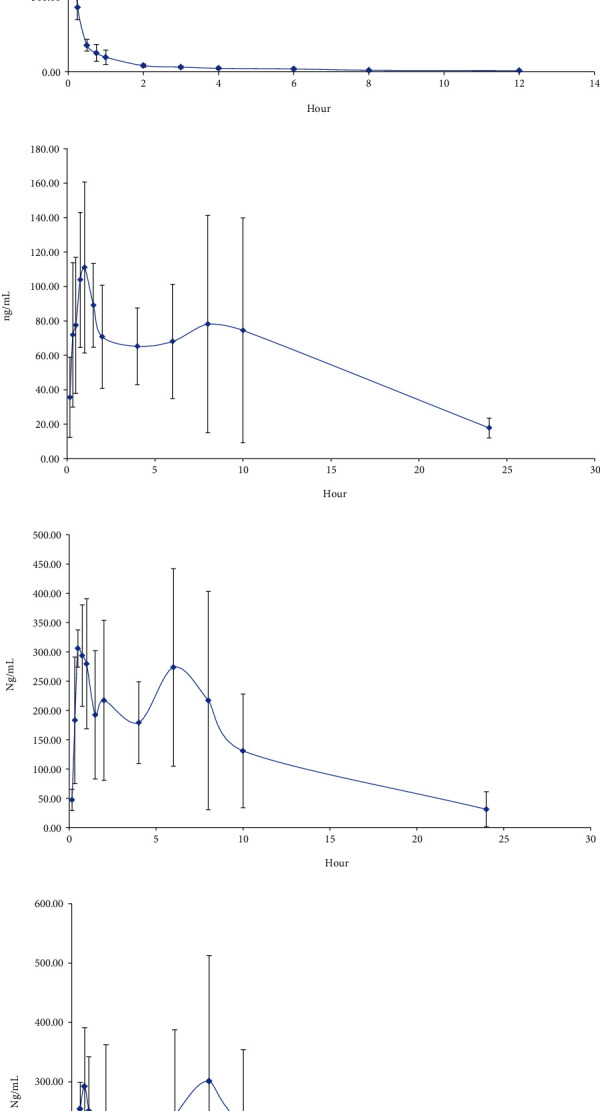
Mean concentration-time curves in rat plasma after oral and intravenous administration of IPA (a) i.v. 5 mg/kg, (b) p.o. 50 mg/kg, (c) p.o. 100 mg/kg, and (d) p.o. 200 mg/kg.

Having successfully validated our LC-MS/MS approach, we next employed it to conduct a pharmacokinetic study of IPA levels in rats that were given IPA either intravenously (5 mg/kg) or orally (50, 100, or 200 mg/kg) in a single dose (*n* = 5 rats/group). Mean IPA plasma concentration-time profiles for these rats are shown in Figure 3, and key pharmacokinetic parameters calculated with a noncompartmental model are compiled in Table 4. In rates intravenously administered IPA, the plasma concentration-time profiles revealed the rapid clearance of this compound, whereas multiple IPA plasma peaks were evident in rats administered this acidic compound via the oral route. The majority of drugs are administered orally, and a range of complex factors influence the dynamics of drug absorption into circulation [20]. Factors such as gastric emptying rate, gastrointestinal motility, and systemic metabolism can all influence oral drug bioavailability and pharmacokinetics [21]. When bioavailability values for these rats were determined as detailed in our Materials and Methods section, the low and intermediate oral IPA doses exhibited respective bioavailability levels of 11.9% and 17.5%, whereas the value for the high IPA dose was outside of our linear analysis range. The primary pharmacokinetic parameters calculated for rats in the 50, 100, and 200 mg/kg oral IPA groups included *T*_max1_ values of 0.9 ± 0.1, 0.6 ± 0.3, and 0.65 ± 0.1, respectively, as well as respective *T*_max2_ values of 6.4 ± 2.6, 6.0 ± 1.4, and 8.80 ± 1.1, and respective half-lives of 10.4 ± 9.8, 5.5 ± 2.3, and 3.6 ± 1.0 h with the AUC_0-24_ rising disproportionately with increasing IPA dose. As such, these data suggest that IPA exhibits nonlinear pharmacokinetic properties in rats across the tested dose range. In addition, there were two peak concentrations at oral administration, suggesting that IPA might be reabsorbed in the rat intestine. Last but not least, the peak concentrations of 100 mg/kg and 200 mg/kg were very similar, suggesting that 100 mg/kg might be the maximum absorption concentration. Thus, 100 mg/kg could be a recommended dosage for isopimaric acid for rats.

## 4. Conclusions

Herein, we successfully developed a reliable, sensitive, and specific LC-MS/MS-based approach to detecting IPA in rat plasma. After validating this assay, we leveraged it to conduct a study of the pharmacokinetics of orally or intravenously administered IPA in rats, providing an important foundation for future research.

## Figures and Tables

**Table 1:** Precision and accuracy of IPA in QC plasma (*n* = 3 days, five replicates per day).

Spiked concentration (ng/mL)	Intraday (ng/mL)	Interday (ng/mL)
10	9.33 ± 1.48	10.3 ± 1.46
400	412.5 ± 1.16	386.3 ± 1.94
2000	2112.4 ± 1.44	1960.7 ± 2.06

**Table 2:** Matrix effects and extraction recovery for IPA and IS in rat plasma (*n* = 5).

Analyte	Spiked concentration (ng/mL)	Absolute matrix effect		Extraction recovery	
		Mean (%)	RSD (%)	Mean (%)	RSD (%)
IPA	10	111.5	11.6	113.4	9.5
	400	119.0	7.1	103.9	4.9
	2000	110.6	9.4	110.2	8.3

**Table 3:** Stability of IPA in rat plasma (*n* = 5).

Spiked concentration(ng·mL^−1^)	Three freeze-thaw	Short-term	Postpreparative	Long-term
	Measured (ng·mL^−1^)	Measured (ng·mL^−1^)	Measured (ng·mL^−1^)	Measured (ng·mL^−1^)
10	10.27 ± 0.56	9.65 ± 0.07	9.56 ± 0.88	10.79 ± 1.46
400	421.8 ± 1.08	407.0 ± 0.34	371.2 ± 1.44	363.5 ± 1.82
2000	1967 ± 0.34	1840 ± 1.6	2012 ± 0.12	1908.3 ± 0.92

**Table 4:** Pharmacokinetic parameters of IPA in rats following p.o or i.v. administration.

PK parameters	i.v.	p.o.		
	5 (mg/kg)	50 (mg/kg)	100 (mg/kg)	200 (mg/kg)
*C*_max1_ (ng·mL^−1^)	1740.1 ± 456.0	83.7 ± 32.9	296.4 ± 75.1	246.23 ± 94.4
*T*_max1_ (h)	0 ± 0	0.9 ± 0.1	0.6 ± 0.3	0.65 ± 0.1
*C*_max2_ (ng·mL^−1^)	/	61.6 ± 46.2	284.9 ± 183.4	286.9 ± 217.2
*T*_max__2_ (h)	/	6.4 ± 2.6	6.0 ± 1.4	8.80 ± 1.1
AUC_(0~24)_	744.7 ± 139.9	886.6 ± 636.2	2604.5 ± 1629.2	3184.7 ± 1721.0
AUC_(0~∞)_ (ng·h·mL^−1^)	818.4 ± 66.2	923.3 ± 613.7	2643.9 ± 1640.0	3193.4 ± 1719.7
CL/F (L·h^−1^)	6.1 ± 0.5	70.3 ± 33.7	59.0 ± 44.0	79.8 ± 42.0
*t*_1/2_ (h)	6.6 ± 5.9	10.4 ± 9.8	5.5 ± 2.3	3.6 ± 1.0
MRT (h)	1.4 ± 0.4	9.3 ± 1.3	7.6 ± 1.3	7.9 ± 0.5

## Data Availability

The statistical data used to support the findings of this study are available from the corresponding author upon request.
